# Biomarkers for Anti-Angiogenic Therapy in Cancer

**DOI:** 10.3390/ijms14059338

**Published:** 2013-04-29

**Authors:** Markus Wehland, Johann Bauer, Nils E. Magnusson, Manfred Infanger, Daniela Grimm

**Affiliations:** 1 Clinic for Plastic, Aesthetic and Hand Surgery, Otto-von-Guericke-University Magdeburg, Leipziger Str. 44, Magdeburg D-39120, Germany; E-Mails: markus.wehland@med.ovgu.de (M.W.); Manfred.infanger@med.ovgu.de (M.I.); 2 Max-Planck Institute for Biochemistry, Am Klopferspitz 18, Martinsried D-82152, Germany; E-Mail: jbauer@biochem.mpg.de; 3 Department of Biomedicine, Pharmacology, Aarhus University, Wilhelm Meyers Allé 4, 8000 Aarhus C, Denmark; E-Mail: nm@farm.au.dk; 4 Medical Research Laboratory, Department of Clinical Medicine, Aarhus University, Nørrebrogade 44, 8000 Aarhus C, Denmark

**Keywords:** cancer, anti-angiogenic therapy, biomarkers, VEGF

## Abstract

Angiogenesis, the development of new vessels from existing vasculature, plays a central role in tumor growth, survival, and progression. On the molecular level it is controlled by a number of pro- and anti-angiogenic cytokines, among which the vascular endothelial growth factors (VEGFs), together with their related VEGF-receptors, have an exceptional position. Therefore, the blockade of VEGF signaling in order to inhibit angiogenesis was deemed an attractive approach for cancer therapy and drugs interfering with the VEGF-ligands, the VEGF receptors, and the intracellular VEGF-mediated signal transduction were developed. Although promising in pre-clinical trials, VEGF-inhibition proved to be problematic in the clinical context. One major drawback was the generally high variability in patient response to anti-angiogenic drugs and the rapid development of therapy resistance, so that, in total, only moderate effects on progression-free and overall survival were observed. Biomarkers predicting the response to VEGF-inhibition might attenuate this problem and help to further individualize drug and dosage determination. Although up to now no definitive biomarker has been identified for this purpose, several candidates are currently under investigation. This review aims to give an overview of the recent developments in this field, focusing on the most prevalent tumor species.

## 1. Introduction

The growth and progression of tumors is crucially dependent on the supply of oxygen and the exchange of nutrients and metabolites with the surrounding tissue. As transport based on diffusion of these molecules is limited to very short distances of less than 3–4 mm only, a tumor exceeding this size needs to develop a vascular system in order to survive. The most important process driving this neovascularization is angiogenesis, the development of new vessels from the existing vascular system [1,2]. Physiologically, angiogenesis is active in normal adults during placenta formation and in wound healing [3,4], but deregulated angiogenesis can occur in diabetes, psoriasis, or rheumatoid arthritis [5]. Most importantly, angiogenesis is also implied in cancer. Starting early during tumor development [6,7] it is an important determinant of tumor aggressiveness and the degree of metastatic spread [8].

On the molecular level, angiogenesis is controlled predominantly by the relatively small family of vascular endothelial growth factors (VEGFs). It comprises VEGF-A (commonly named VEGF), VEGF-B, VEGF-C, VEGF-D, VEGF-E, and placenta growth factor (PlGF) [9–12]. Moreover, angiogenesis can be induced also via alternative pathways by other soluble factors such as fibroblast growth factor 1 and 2 (FGF1 and FGF2), angiopoietin or ephrin A1 and A2 [13,14]. While basal (blood) VEGF levels are necessary to sustain an intact vascular system [15], many tumors are characterized by elevated secretion of various VEGF isoforms [16]. This is considered to be a reaction of the cancer cells to a hypoxic and growth-factor rich environment [17–19] they are exposed to due to their high proliferation rate. The elevated VEGF levels may stimulate endothelial cells of nearby blood vessels to develop new vessels in order to supply the tumor with nutrients and oxygen and thus support its further growth [20–25].

The VEGF proteins are ligands for three tyrosine kinase receptors: VEGF receptor 1 (VEGFR-1, also called Flt-1), VEGF receptor 2 (VEGFR-2), and VEGF receptor 3 (VEGFR-3) [26–28]. Vascular endothelial cells predominantly express VEGFR-1 (with VEGF-A, VEGF-B, and PlGF as ligands) and VEGFR-2 (with VEGF-A as its main ligand [29]), whereas VEGFR-3 (binding VEGF-D and VEGF-E [30]) is mostly found in lymphatic endothelial cells controlling lymphangiogenesis, but can also be expressed in tumor vessels or chronic inflammatory wounds [16,31,32].

VEGF-A is the central factor in the promotion and regulation of tumor angiogenesis, VEGF signaling is mainly mediated by VEGFR-2 [33,34], while VEGFR-1 is believed to act as a decoy receptor, controlling VEGF availability [35,36]. VEGF-A/VEGFR-2 signaling plays an important role in both, physiological and pathophysiological processes, including burn injury [37], wound healing [38,39] and tumor angiogenesis. Importantly, it has been demonstrated as well that VEGF-A is a survival factor for endothelial cells [20,40–43].

## 2. Anti-Angiogenic Therapy

Considering the outstanding importance of angiogenesis for tumor growth and survival in general and the role of the VEGF-A/VEGFR-2 signaling system in particular, several drugs have been developed, which interfere with different angiogenic molecules. In principle, three different modes of action are possible: interception of the VEGF ligand, blockade of the VEGFR, or interruption of the intracellular VEGFR-mediated signaling.

Examples for drugs targeting the VEGF-A ligand are Bevacizumab and Aflibercept. Bevacizumab is a VEGF-binding humanized recombinant antibody, which inhibits the VEGF-VEGFR-interaction [44]. In the clinical context it has been used in lung [45–48], breast [49–53], colon [54–58], renal [59,60], gastric [61], pancreatic [62,63], and prostate cancer [64], as well as melanoma [65]. Aflibercept is a fusion protein of the human Fc part of IgG1 and the extracellular domain of VEGFR. As such it is able to quench VEGF-A and -B, and PlGF-1 and 2, effectively removing the soluble ligands from the VEGF-VEGFR-cascade. So far Aflibercept has been applied in ovarian [66], colorectal [67], lung [68], metastatic gynecologic soft-tissue [69], and urothelial metastatic transitional cell cancer [70], as well as melanoma [71] and glioblastoma [72].

Ramucirumab, a human antibody specific for the extracellular ligand-binding domain of VEGFR-2, belongs to the class of VEGFR-blocking drugs [73]. Ramucirumab has been used in studies for a multitude of different cancer types and has shown the best results for stable disease (only minor increases or decreases in tumor size) in renal, uterine, colorectal, and ovarian carcinoma [74].

**Figure 1:**
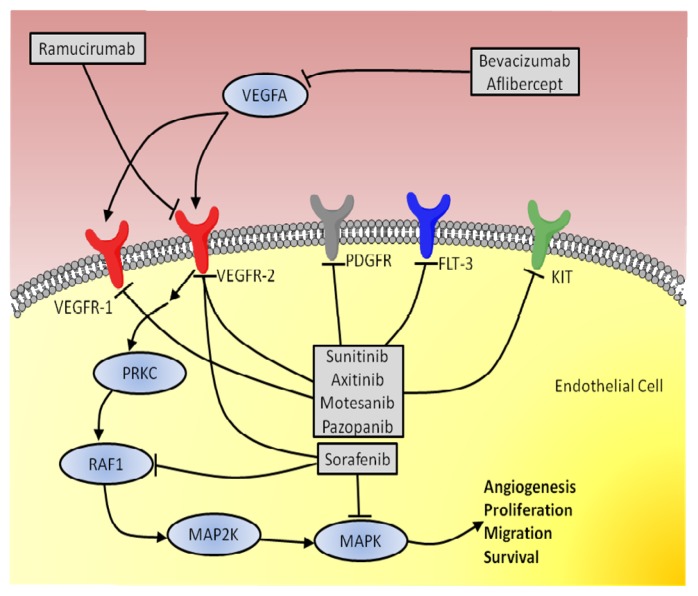
Overview of representative anti-angiogenic drugs and their targets in the angiogenic pathway. FLT-3: Fms-like tyrosine kinase 3; KIT: Mast/stem cell growth factor receptor; MAP2K: Mitogen-activated protein kinase kinase; MAPK: Mitogen-activated protein kinase; PDGFR: Platelet derived growth factor receptor; PRKC: Protein kinase C; RAF1: Proto-oncogene c-RAF; VEGFA: Vascular endothelial growth factor A; VEGFR-1: Vascular endothelial growth factor receptor 1; VEGFR-2: Vascular endothelial growth factor receptor 2.

Small-molecule tyrosine kinase inhibitors (TKIs) inhibit ATP binding to the tyrosine domain of VEGFRs and therefore interrupt the VEGFR signal transduction. The most prominent members of this class of drugs are Sunitinib and Sorafenib, which are primarily used in renal [75] and gastrointestinal stromal tumors [76] or renal [77] and hepatocellular cancer [78], respectively. Further members include Motesanib, which is used in lung [79] and medullary thyroid cancer [80], or Pazopanib, which is approved for renal cell cancer and soft tissue sarcomas [81] (see Figure 1 for a brief overview).

However, although many studies seem to indicate a modest benefit of anti-angiogenic therapy, it generally suffers from a high variability in the response by the individual patient. Possible reasons for this observation may be the general capability of endothelial cells to form new vessels independently of an enhancement of the VEGF-A/VEGFR-2 signaling system. In a series of *in vitro* experiments, our group could demonstrate that cellular changes [82,83] induced by culturing endothelial cells under simulated microgravity trigger some cells to form tubes, which resemble the intima of blood vessels [43,84,85]. This suggests that some types of cancer cells may activate a mechanism inducing neighboring endothelial cells to provide a supply of oxygen and nutrients to a tumor when the VEGF/VEGFR signaling system is switched off by drugs. Therefore, it would be an important milestone to identify factors, which indicate the tendency of some tumor-subtypes in developing evasive strategies against anti-angiogenesis treatment. Knowledge of such factors will allow the prediction of and the adequate reaction to these effects. Hence a more individualized treatment of any patient could enhance the success of the therapy.

## 3. Biomarkers

Cancer cells are usually classified by biomarkers. A biomarker, as defined by the NIH, is “a characteristic objectively measured and evaluated as an indicator of normal biologic processes, pathogenic processes, or pharmacologic responses to a therapeutic intervention” [86]. Several types of biomarkers can be distinguished. Prognostic biomarkers help estimating the overall disease outcome, independent from therapy [87]. Predictive biomarkers on the other hand provide information about the response or survival of a certain patient under a specific treatment prior to therapy [88]. Furthermore, biomarkers can also have screening, diagnostic, pharmacodynamic, and safety-related properties or act as surrogate parameters. The use of biomarkers might therefore be a way to circumvent the drawbacks of indiscriminate anti-angiogenic therapy by enabling physicians to select patients with the highest likelihood for a positive response to a treatment. This review will focus on recent developments in biomarkers for anti-angiogenic therapy in the most prevalent cancers.

### 3.1. Biomarkers in Colorectal Cancer

Colorectal cancer is the second most frequent cause of death in North America and Europe with about 600,000 deaths and a rate of approximately 1.2 million new cases per year worldwide [89]. At the moment, no confirmed biomarkers are known allowing the prediction of anti-angiogenic therapy efficacy for this cancer. It was found in clinical trials that the survival benefit from adding Bevacizumab to standard chemotherapy was neither determined by K-ras, BRAF, or p53 mutation status nor by VEGF, p53 or thrombospondin 2-expression [90,91]. For that reason it is important to find new candidates to identify suitable colorectal tumor patients for VEGF-targeted therapeutic approaches.

#### 3.1.1. Circulating Biomarkers

Blood is a relatively easily available material for the analysis of biomarkers and has a great potential for this application. Several studies have analyzed circulating molecules for their potential as predictive biomarkers for colorectal cancer. Cetin *et al.* [92] found that in a cohort of patients treated with FOLFIRI or XELOX in combination with Bevacizumab, serum LDH and neutrophil levels higher than the upper limit of normal (ULN) were independent predictors of short term survival. With a similar therapeutic regimen, Kopetz *et al.* [93] have screened a total of 37 plasma cytokines and circulating angiogenic factors for their use as biomarkers for treatment response or resistance to a FOLFIRI/Bevacizumab therapy. From this panel, elevated IL-8 levels at baseline were linked to a shorter progression-free survival (PFS).

Angiopoietin-2 is another potentially valuable circulating biomarker. It has been proposed to be involved in VEGF function and vascular remodeling. Interestingly, low serum angiopoietin-2-levels were found to be associated with a high overall survival (OS) of >90% after 18 months and a better response rate to anti-angiogenic therapy of >80% compared to high serum angiopoietin-2-levels in a study analyzing patients receiving Bevacizumab in combination with different chemotherapeutic regimens (FOLFOX, FOLFIRI, XELOX, XELIRI) [94].

Finally, circulating endothelial cells (CEC) have been found to predict the response to Bevacizumab-therapy in colorectal cancer. Blood from patients receiving FOLFOX4 and Bevacizumab was analyzed for endothelial cells before and during treatment. It could be shown that no correlation existed between the CEC levels and the outcome in a FOLFOX4 alone control group. However, CEC proved to be a strong indicator for the outcome of the Bevacizumab-based therapy. Patients with less than 65 CEC/4 mL blood at baseline (as determined by the CellSearch system), had a significantly longer median PFS and OS than patients with 65 CEC/4 mL or more. In addition, a low proportion of CXCR4-positive CEC of below 20% at baseline was also correlated to significantly longer PFS and OS [95,96]. These findings were confirmed by another study, which showed that a total number of 40 CEC/mL or less was connected to longer PFS [97]. The same group further investigated the course of CEC levels over the duration of treatment and the role of the fraction of apoptotic cells among them. They found that increases of both CEC and the apoptotic CEC subpopulation at the 6th cycle of Bevacizumab-based therapy were statistically significant indicators for better PFS [98].

#### 3.1.2. Genetic Biomarkers

Genes involved in angiogenesis show a relatively high level of variation, ranging from silent SNPs to functional polymorphisms. The latter were tested in the *VEGF*, *KDR*, *IL6*, *CXR1* and *-2*, *P53, MMP2*,*-7*,*-9*, and *ICAM* genes of patients with metastatic colorectal cancer receiving FOLFOX or XELOX with Bevacizumab using genomic DNA from peripheral blood. The IL-6 G-174C and P53 codon 72 polymorphisms were found to be correlated to a positive response to Bevacizumab therapy. Furthermore, PFS was significantly associated with MMP9 C-1562T and CXCR-1 G + 2607C [99].

In another study, the Nordic ACT trial (Bevacizumab + FOLFOX, XELOX; FOLFIRI, or XELIRI), the VEGFR-1 319 C/A SNP has been identified as significantly associated with response to Bevacizumab. Objective response rates differed significantly between the three genotypes (CC = 36% *vs.* CA = 40% *vs.* AA = 56%, *p* = 0.048, or CC + CA = 39% *vs.* AA = 56%, *p* = 0.015), indicating that the A-allele exerts a strong beneficial effect [100].

CD133, a trans-membrane protein isolated from colorectal cancer stem cells [101,102], has biomarker properties as well. Two SNPs have been identified as being useful in the prediction of PFS and OS in patients treated with FOLFOX/Bevacizumab or XELOX in first line [103]. Patients carrying either CC in both rs2286455 and rs3130 SNPs or a combination of CT with CT or TT exhibited a doubled PFS (16.5 months *vs.* 8.4 months, *p* = 0.010) after treatment with FOLFOX/Bevacizumab as compared to the rest.

#### 3.1.3. Physiologic Biomarkers

Hypertension is a very common side effect of VEGF inhibitor medications, with general incidence rates of about 20% and grade 3 hypertension percentages of approximately 11% [56, 104]. Hurwitz *et al.* concluded in their recent paper that early treatment-related blood pressure increases do not predict clinical benefit from Bevacizumab based on PFS or OS outcomes. BP increases do not appear to have general prognostic importance for patients with advanced cancer [104]. Although the exact mechanism of hypertension induction by anti-angiogenic treatment is unknown, it is hypothesized that VEGF signaling influences NO-synthase activity. VEGF inhibitors might therefore reduce NO-production, leading to increased vasoconstriction and ultimately hypertension, which would be an indicator for a successful inhibition of angiogenesis [105]. This idea has been confirmed by different studies. Scartozzi *et al.* observed that Bevacizumab-induced grade 2–3 hypertension in patients receiving Irinotecan, Fluorouracil, and Bevacizumab was significantly associated with improved PFS (14.5 months *vs.* 3.1 months, *p* = 0.04) [106]. Österlund *et al.* described that hypertension was associated with prolonged PFS (10.5 months *vs.* 5.3 months, *p* = 0.008) and OS (25.8 months *vs.* 11.7 months, *p* < 0.001) in patients treated with Bevacizumab-containing chemotherapy [107]. Development of hypertension within three months was identified as an independent prognostic factor and no relation of hypertension to thromboembolic complications could be detected. In a retrospective analysis of patients with a therapeutic regimen of FOLFIRI, FOLFOX, XELOX, XELIRI, or FOLFOXIRI together with Bevacizumab, De Stefano *et al.* observed an induced grade 2–4 hypertension in 17.6% of the cases. Of the patients with induced arterial hypertension, 84.6% achieved a complete or partial response, whereas patients without these side effects only had 41.9% (*p* = 0.006). In addition and comparable to the other studies, hypertension was associated with improved PFS (15.1 months *vs.* 8.3 months, *p* = 0.04) [108].

### 3.2. Biomarkers in Breast Cancer

Among women, breast cancer is the malignancy with the highest occurrence at a rate of about 23% and a total number of approximately 1.4 million new cases per year worldwide. Furthermore, with about 460,000 cases per year it is also the most frequent cause of death due to cancer [89]. Unfortunately, so far anti-angiogenic therapy in general had only limited success. Most clinical studies showed no benefit in OS and PFS, the FDA withdrew its approval of Bevacizumab as a drug against metastatic HER2-negative breast cancer, and Sunitinib has failed altogether [109]. However, although anti-angiogenic therapy does not seem to be an option for every breast cancer patient, it is interesting to note that in each trial were patients who benefitted strongly from this regimen. So far no pattern has been identified on how to select these individuals, but efforts are ongoing to discover biomarkers for this purpose.

#### 3.2.1. Circulating Biomarkers

In a study with combined Bevacizumab/Vinorelbine chemotherapy Burstein *et al.* could demonstrate that baseline VEGF plasma levels were associated with the time to progression (TTP). Patients with VEGF concentrations >32.6 pg/mL had a median TTP of 3.7 months, whereas patients with VEGF levels <32.6 pg/mL had a median TTP of 9.3 months (*p* = 0.003) [110]. In addition, in locally advanced breast cancer preoperatively treated by Docetaxel with or without Bevacizumab, low baseline serum concentrations of both VCAM-1 and E-selectin were significant (*p* = 0.033 and *p* = 0.035, respectively) predictors of clinical response in the form of operability [111]. Burstein *et al.* measured the VEGF, soluble VEGFR-2 (sVEGFR-2), soluble VEGFR-3 (sVEGFR-3), and soluble KIT (sKIT) plasma levels at baseline and during therapy in patients receiving Sunitinib after chemotherapy with and Anthracycline or Taxane. They found a trend for a connection of decreasing sVEGFR-3 levels (≥20%) with longer OS (*p* = 0.07). In addition, decreases of sKIT levels by ≥50% were significantly associated with longer TTP (*p* < 0.001) and OS (*p* = 0.0194) [112]. Finally, CEC proved to be predictors of anti-angiogenic therapy success in a cohort of patients receiving metronomic, *i.e.*, repeated, low-dosed, chemotherapy in conjunction with Bevacizumab. A significant association of high baseline levels of CEC with overall response (*p* = 0.02), clinical benefit (*p* = 0.01), and improved PFS (*p* = 0.04) was observed in this study [113].

#### 3.2.2. Genetic Biomarkers

So far, only polymorphisms in the VEGF and VEGFR2 genes have been analyzed for their potential to serve as a biomarker for anti-angiogenic therapy in breast cancer. Schneider *et al.* did a retrospective study on the ECOG2100 cohort, investigating five SNPs for VEGF and two for VEGFR2. They found that VEGFR2 polymorphisms did not show any influence on OS or any other clinical parameter. Two VEGF genotypes, on the other hand, VEGF-2578 AA and VEGF-1154 AA, were significantly associated with improved OS (*p* = 0.023 and *p* = 0.001, respectively) in the Paclitaxel/Bevacizumab combination arm of the study [114].

#### 3.2.3. Physiologic Biomarkers

The role of anti-angiogenic therapy induced hypertension is subject of debate, as currently there are contradicting results found in the literature. Beside identifying two VEGF-SNPs as candidate biomarkers, Schneider *et al.* also observed in the same study that patients who developed grade 3–4 hypertension had an improved median OS of 38.7 months *vs.* 25.3 months of normotensive patients (*p* = 0.002) [114]. In contrast to this, a meta-analysis of clinical outcome under anti-angiogenic medication (including the RIBBON-1 trial, comparing Bevacizumab + chemotherapy *vs.* Bevacizumab + placebo) did not show a prognostic value of early hypertension [115]. Further investigations are needed to clarify this situation.

#### 3.2.4. Tissue Biomarkers

Blood levels of VEGF and related molecules can be influenced by many different additional factors besides cancer and may therefore be misleading or difficult to interpret in an analysis of their predictive value. Hence it might be a better approach to directly determine the expression of candidate genes or proteins inside the tumor tissue itself. This, of course, poses some problems, like easy availability of ideally multiple samples over the course of the therapy.

Fountzilas *et al.* immunohistochemically investigated a panel of biomarker candidate proteins in patients receiving a first line Paclitaxel/Bevacizumab therapy against metastatic breast cancer. They found that high intra-tumoral expression of VEGFR-3 was associated with clinical response, whereas VEGFR1 overexpression was an indicator for poor survival [116].

### 3.3. Biomarkers in Thyroid Cancer

Although representing only a small portion of cancer cases with an incidence of about 213,000 new cases (which equates to approximately 1.7%) and a mortality of a bit over 35,000 cases per year worldwide, thyroid cancer is the most frequent malignant endocrine tumor [89,117]. For a long time radio-therapy was the classical way to fight this disease [118]. Besides certain subtypes, such as the progressive or advanced medullary (MTC), or the differentiated (DTC) thyroid cancers [119,120], especially distantly metastatic tumors are still difficult to treat with the traditional therapies [121,122]. Earlier studies have shown that targeting endothelial cells in SCID mice bearing a follicular thyroid ML-1 tumor by the tyrosine kinase inhibitor PTK787/ZK222584 reduced tumor growth and vascularization [123]. In addition, an Angs/Tie-2 system dysfunction was suggested to play an important role in thyroid tumorigenesis. The decrease of the concentration of the angiogenesis inhibitor Ang-1 in serum was a useful additional biomarker for the presence of thyroid cancer [124]. Liang *et al.* demonstrated that MMP2, PTTG, VEGF-C, CXCR4 and bFGF are potential cellular tumor markers for identifying thyroid cancer with greater risk for metastasis. The authors suggest that the combination of the angiogenic factors VEGF-C and bFGF favors progression in metastatic thyroid carcinoma [125]. Thus, treating thyroid cancer with the help of pharmacological inhibitors of angiogenesis was considered to be a promising new way of fighting thyroid cancer [126]. Subsequently, over the course of the last five years, the option of anti-angiogenic treatment has been explored [127–133]. The results were encouraging, but a considerable variation in the response of the various patients was observed. Therefore, predictive biomarkers are sought, which could indicate the success of an anti-angiogenic treatment in advance.

#### 3.3.1. Circulating Biomarkers

Broutin *et al.* showed in 2011 [134] that cytokines are possible biomarkers for the tumor response towards Sunitinib treatment of in medullary thyroid carcinomas (MTC). A significant decrease of tumor growth and angiogenesis was observed after Sunitinib therapy in a mouse model, which is associated with significantly decreased serum IL-8 levels. In parallel, the serum of 27 patients with MTC showed significantly increased serum concentrations of IL-8 compared to the healthy donor population [134]. In addition, we found that IL-8 gene expression is involved in human thyroid cancer cell tumor formation [135]. Therefore, IL-8 appears very interesting as a therapeutic target and as a clinical biomarker for the success of an anti-angiogenic treatment.

Bass *et al.* analyzed a cohort of patients with progressive advanced thyroid cancer receiving Motesanib as anti-angiogenic therapy over a period of 48 weeks [136]. During this time, the group determined serum and plasma concentrations of sVEGFR1 and -2, PlGF, VEGF, bFGF, sKIT, sVCAM-1, angiopoietin-1 and 2, and enzyme activities of caspases-3 and -7. It was found that patients with baseline VEGF levels below 671 pg/mL had a significantly better PFS than patients with higher baseline VEGF concentrations (*p* = 0.0007). Furthermore, the study could show that not only absolute serum concentrations or activities but also their changes during treatment were a useful indicator for patient response to therapy. Increases of PlGF by more than 4.7 fold (*p* < 0.0001) and of caspase 3/7-activity by more than 2.1-fold (*p* < 0.0001) as well as changes by less than −1.6-fold of sVEGFR2 (*p* < 0.0001) were independent predictors enabling investigators to separate responders from non-responders [136]. In another study, Sorafenib was applied in advanced iodine-refractory differentiated thyroid cancer. The investigators analyzed serum thyroglobulin (Tg) levels and found that both Tg baseline levels as well as Tg response were useful in predicting the clinical outcome of anti-angiogenic therapy with Sorafenib [137]. Furthermore, Sorafenib and Sunitinib have been shown to be effective in patients with widely metastatic, progressive differentiated thyroid cancer. Logarithmic thyroglobulin (Log Tg) significantly correlated with response to this treatment [138]. Now, serum thyroglobulin levels are suggested to have a value as a surrogate marker of response. So far, Tg had been used as a diagnostic tool [139] or as a predictor of recurrence after thyroidectomy [140,141].

#### 3.3.2. Tissue Biomarkers

Shaik and coauthors described a subtype of human papillary thyroid cancer (PTC), which is resistant to therapy with VEGF receptor 2 (VEGFR2)-inhibitor. In these poorly differentiated PTC cells, the beta-transducin-repeat-containing protein inhibits cell migration and decreases sensitivity to Sorafenib [142]. In various thyroid carcinomas, hypoxia-inducible factor-1alpha expression was found to be increased [143]. Zerilli *et al.* demonstrated that hypoxia-inducible factor-1alpha is expressed in papillary thyroid carcinomas and is not only regulated by hypoxia but also by the BRAF(V600E)-mediated signaling pathway [143]. BRAF(V600E) (serine/threonine-protein kinase B-raf) is an oncogene linked to angiogenesis. Its presence in a cancer cell favors angiogenesis [144]. Besides the two preceding proteins, a number of proteins, especially membrane proteins, may be indicators of successful and un-successful angiogenic therapy [145]. Therefore, our group applies the methods of free-flow IEF and mass spectrometry to screen and evaluate as many proteins of thyroid cancer cells as possible in comparison to their behavior [146–149].

### 3.4. Biomarkers in Renal Cancer

Renal cancer is among the 10 most frequently occurring cancers in western countries and accounts for more than 100,000 deaths worldwide per year [150]. Renal cell carcinomas (RCC) comprise approximately 90% of renal cancers with the most common histological subtype being clear cell RCC (ccRCC) accounting for 80% of the cases. Up to 70% of patients are presented with localized disease, and approximately one third of these will relapse with metastatic RCC following radical or partial nephrectomy. This clearly indicates the need for tools to maximize the benefit of drug treatment and evaluate the risk of relapse on an individual basis. Today, classification of RCC is largely based on morphology and despite the emergence of promising prognostic biomarkers in RCC, none have been routinely applied in the clinic. Furthermore, no predictive biomarkers are used to identify patients who might benefit from a given treatment.

Knowledge of ccRCC biology has led to a number of anti-angiogenic systemic therapies targeting VEGF either directly by inhibiting antibodies or via targeted tyrosine kinase inhibitors. First line adjuvant therapies include Sunitinib and Pazopanib which are effective in 70%–80% of cases. There are no biomarkers available that may discriminate between patients who will benefit from this treatment and those who will not.

#### 3.4.1. Circulating Biomarkers

Plasma VEGF and soluble VEGFR-2 (sVEGFR-2) has been tested as predictive markers of anti-angiogenic treatment in phase III Treatment Approaches in Renal Cancer Global Evaluation Trial (TARGET). In this study high baseline levels of VEGF were associated with poor prognosis but baseline sVEGFR-2 and changes in VEGF or sVEGFR-2 could not predict the response to Sorafenib [151]. In phase II trials using Sunitinib or Pazopanib significant changes in sVEGFR-2 levels were demonstrated in patients showing objective tumor response to treatment compared to patients with stable or progressive disease [152,153]. In a recent study of an unselected population of advanced kidney cancer patients receiving Sunitinib, serum levels of circulating neutrophil gelatinase associated lipocalin (NGAL) and VEGF were strongly associated to an improved progression free survival in both univariate and bivariate analyses and performed better than the Motzer score considered golden standard [154]. NGAL is upregulated in cells during “stress” and is tightly coupled to matrix metalloproteinase 9 (MMP-9), involved in the degradation of the extracellular matrix [155], making these proteins relevant for further analyses.

High levels of CAIX protein was shown to correlate to the responsiveness of interleukin (IL)-2 with 78% of patients responding to IL-2 showing high expression (>85% tumor cells) of CAIX protein in tumors compared to 51% in non-responders. The value of CAIX as a predictive marker is currently under investigation [156,157]. Similarly, HIF-1 alpha expression has been implicated as a potential prognostic marker where high (>35%) tumor-immunostaining levels were shown to correlate to shorter survival [158]. In addition, HIF-2 alpha expression was correlated to a beneficial responsive to Sunitinib in 43 ccRCC samples [159]. Other promising protein markers include tumor-associated B7-H1 and insulin-like growth factor II mRNA binding protein 3, both of which have been independently validated and add value to existing nomograms in RCC [160,161], but have not found their way into the clinic. Recent studies suggest that in addition to using only single biomarkers it might be more promising to screen a whole panel of cytokines and angiogenic factors (CAFs). Zurita *et al.* were able to identify a candidate CAF signature that may help predict PFS benefit from Sorafenib treatment in patients suffering from metastatic RCC [162,163].

#### 3.4.2. Genetic Biomarkers

##### Single Nucleotide Polymorphisms

Genome-wide association studies have reported SNPs that may increase the risk of developing RCC [164,165]. A large study comprising 397 patients with RCC treated with Pazopanib addressed the response to TKI therapy [166]. 27 SNPs in 13 genes were reported including genes related to angiogenesis VEGF, IL-8 and fibroblast growth factor 2. Two polymorphisms in IL-8 were significantly associated to a shorter PFS of 27 weeks compared to the wild type genotype (48 weeks). Notably, IL-8 has recently been suggested as to be involved in resistance to TKIs [167]. In another study the response and toxicity of Sunitinib were evaluated in patients with ccRCC. Two VEGFR-3 SNPs were associated with reduced PFS, a variant of CYP3A5*1 was associated with increased toxicity, however, the IL-8 variants described above were not found in this study [168]. Interestingly, a third study found that SNPs in CYP3A5 increased survival [169], indicating little concordance between these studies. To some extent these disparate observations may be explained by sample number, indicating the need for large studies to increase the statistical power. Perhaps an even greater challenge arises from the heterogeneity found within individual tumors. This point was demonstrated using multi-region genetic analysis, showing that up to two thirds of mutations found in one region of the tumor were not present in other regions of the same tumor, suggesting that both favorable and unfavorable conclusions can be made depending on the specific specimen sampling [170]. Clearly, heterogeneity is a feature of all cancers, but to what extent these differences impact the tumor phenotype and therapeutic targets is unknown. However, mRNA profiling and expression of protein biomarkers are likely more robust to these changes as they are upstream of these events.

### 3.5. Biomarkers in Prostate Cancer

Among men prostate cancer (PC) is the second most frequently occurring cancer with about 900,000 new cases and approximately 260,000 deaths per year worldwide. It also has by far the highest worldwide 5-year prevalence of roughly 24% [89]. In USA and Europe, PC is estimated to account for 25% of cancer diagnoses in males and 9% of cancer related deaths [171,172]. The diagnosis of PC includes measurement of serum prostate-specific antigen (PSA), rectal examination and morphological/histological evaluation of needle biopsy. The treatment of prostate cancer includes prostatectomy and radiation, while androgen withdrawal is used to delay the progression of metastatic disease [173]. However, over time tumors become resistant to androgen deprivation and develop into castrate resistance PC (CRPC) with high morbidity and mortality. Therefore, anti-angiogenic treatment was considered an alternative way to fight PC. But, similar to breast cancer, anti-angiogenic therapy of prostate cancer has shown only moderate to disappointing effects, with little or no improvement in OS resulting from addition of Bevacizumab to a standard Docetaxel and Prednisone therapeutic regimen [64]. Nevertheless, trials with different anti-angiogenic drugs are still ongoing and biomarkers for the assessment of therapeutic efficiency are needed.

### 3.6. Future Developments

So far, most analyses of possible biomarker candidates for the prognosis and prediction of anti-angiogenic therapy have been conducted with a rational approach (Table 1), concentrating on molecules which are more or less directly involved in angiogenic pathways. As summarized in this article, many factors, initially expected to yield clear results, proved to be not as robust for this purpose, with the most prominent example being VEGF. Many candidate parameters suffer from poor reproducibility across different tumor types and there are still not enough studies comparing the same markers in different cancers [174]. Therefore it might be necessary to think more “outside the box” and to employ a wider, assumption-free strategy by using the increasingly easier available techniques for genomic and proteomic analysis of the samples. Gene expression profiling, preferably of endothelial cells originating from the tumor tissue, might provide further insight into the different types of tumor vasculature and help select the appropriate medication [175]. Investigating the cancer cell proteome and secretome might also lead to the identification of new, so far neglected molecules which are more effective than the “classical” candidates [176,177]. Moreover, decisions about the type of therapy may in future be based on a multitude of parameters. A profile comprising a number of different potential markers could help to predict the benefit of an anti-angiogenic therapy more robustly and reliably than a single biomarker, as attempted with CAF screening [162].

## 4. Conclusions

Anti-angiogenic therapy generally suffers from a high variability in the response by the individual patient. In order to select patients with the highest likelihood for a positive response to such a treatment, the availability of reliable predictive biomarkers for anti-angiogenic therapy will be a key factor. Although there are some promising preliminary results, no general or cancer-specific biomarker has yet emerged, which could help select patients with a positive prognosis for anti-angiogenic therapy. For its future it is therefore of vital importance to conduct larger systematic trials to translate the preclinical data into clinically usable systems and to switch from unselective therapy to a more individual drug selection based on the patients’ predispositions.

## Figures and Tables

**Table 1:** Prognostic biomarkers for anti-angiogenic therapy

Type	Parameter	Cancer	Finding	References
Circulating	Serum LDH and neutrophil levels	Colon	LDH and neutrophil levels > ULN predict short survival	[92]
	IL-8	Colon	Elevated IL-8 linked to shorter PFS	[93]
	Angiopoietin-2	Colon	low serum levels associated with high OS	[94]
	Circulating endothelial cells (CEC)	Colon	CEC < 65/4mL associated with longer PFS and OS	[95–98]
		Breast	High baseline levels associate with improved OR and PFS	[113]
	VEGF plasma levels	Breast	<32.6 pg/mL associated with longer median TTP	[112]
		Thyroid	baseline concentrations ≤671 pg/mL associated with improved PFS	[136]
		Renal	High baseline levels associated with poor prognosis	[151]
	PlGF and sVEGFR2 plasma levels and caspase 3/7 activity	Thyroid	Changes by more than 4.7, −1.6, and 2.1-fold, respectively, indicate response	[136]
	sVEGFR2 plasma levels	Renal	Significant changes associated with objective tumor response	[152, 153]
	Serum NGAL and VEGF levels	Renal	Associated with improved PFS	[154]
	VCAM-1 and E-selection serum levels	Breast	Low levels associated with improved clinical response	[111]
	sKIT plasma level	Breast	Decrease ≥ 50%associated with longer TTP	[112]
	Serum Tg levels	Thyroid	Predictor for clinical outcome	[137, 138]
	CAF screen	Renal	Predictor for PFS benefit	[162]
Genetic	MMP9 C-1562T and CXCR-1 G + 2607C	Colon	Associated with longer PFS	[99]
	VEGFR-1 319 C/A	Colon	A-allele has strong beneficial effect	[100]
	CD133 rs2286455, rs3130, and rs2240688 SNPs	Colon	Associated with PFS and OS	[103]
	VEGF-2578 AA and VEGF-1154 AA	Breast	Associated with improved OS	[114]
	ccB subtype	Renal	Associated with poor prognosis	[170]
	VEGFR-3 and CYP3A5^*^1 SNPs	Renal	Associated with increased Sunitinib toxicity	[168]
Physiologic	Hypertension	Colon	Associated with improved PFS	[106–108]
		Breast	Associated with improved OS	[113]
Tissue	Tumor VEGFR-3 expression	Breast	Overexpression associated with poor survival	[115]
	Tumor BTRC expression	Thyroid	Mediates Sorafenib-resistance	[142]

## Abbreviations

AMACR: Alpha-methylacyl-CoA racemase
Ang: Angiopoietin
ATP: Adenosine triphosphate
BRAF: Raf murine sarcoma viral oncogene homolog B1
CAF: Cytokine and angiogenic factor
CAIX: Carbonic anhydrase IX (CAIX)
CEC: Circulating endothelial cells
CD: Cluster of differentiation
CR: Castration resistance
CT: Computer tomography
CTC: Circulating tumor cells
CXCR4: CXC motif, Chemokine receptor type 4
DNA: Deoxyribonucleic acid
DTC: Differentiated thyroid cancer
FDA: Food and Drug Administration
FDG: ^18^F-fluorodeoxyglucose
FGF: Fibroblast growth factor
FLT-3: Fms-like tyrosine kinase 3
Fluorouracil: Folinic acid
FOLFIRI: Irinotecan combination therapy
FOLFOX4: Oxaliplatin combination therapy
HER2: Human Epidermal Growth Factor Receptor 2
HIF: Hypoxia-inducible transcription factor
ICAM: Intercellular adhesion molecule
IEF: Isoelectric focusing
IgG: Immunoglobulin G
IL: Interleukin
K-ras: Kirsten rat sarcoma viral oncogene homolog
KDR: Kinase insert domain receptor
KIT: Mast/stem cell growth factor receptor
LDH: Lactate dehydrogenase
MAPK: Mitogen-activated protein kinase
MAP2K: Mitogen-activated protein kinase kinase
mCRPC: Metastatic castration-resistant prostate cancer
MMP: Matrix metalloproteinase
MRI: Magnetic resonance imaging
MTC: Medullary thyroid carcinoma
NGAL: Neutrophil gelatinase associated lipocalin
NIH: National Institutes of Health
NO: Nitric oxide
OS: Overall survival
PC: Prostate cancer
PET: Positron emission tomography
PFS: Progression-free survival
PlGF: Placenta growth factor
PRKC: Protein kinase C
PSA: Prostate-specific antigen
PTC: Papillary thyroid cancer
PTTG: Pituitary Tumor-Transforming Gene 1-Interacting Protein
RAF1: Proto-oncogene c-RAF
RCC: Renal cell carcinomas
RIBBON-1: Regimens in Bevacizumab for Breast Oncology-1
RNA: Ribonucleic acid
SCID: Severe combined immunodeficiency
SNP: Single Nucleotide Polymorphisms
TARGET: Treatment Approaches in Renal Cancer Global Evaluation Trial
Tg: Thyroglublin
TKI: Tyrosine kinase inhibitor
TNM: Tumor Node Metastasis system
TTP: Time to progression
ULN: Upper limit of normal
VCAM: Vascular cell adhesion molecule
VEGF: Vascular endothelial growth factors
VHL: Von Hippel-Lindau tumor suppressor
XELIRI: Capecitabine and Irinotecan combination therapy
XELOX: Capecitabine and Oxaliplatin combination therapy.
